# MR-Guided Adaptive Radiotherapy in Lung Cancer: Current Landscape and Future Directions

**DOI:** 10.3390/cancers18172819

**Published:** 2026-09-01

**Authors:** Abrahams Ocanto, Macarena Teja, Daniela Gonsalves, Walter Vásquez, Inmaculada Navarro-Domenech, Marta Moreno-Jimenez, Gloria Guardia, Dulce Urias, Scarlet Crespo, Mariana Temes, Nicolly Hernández, Suresh Senan, Drew Moghanaki, Rosa Morera, Ignacio Azinovic, Javier Luna, Felipe Couñago

**Affiliations:** 1 Department of Radiation Oncology, San Francisco de Asís University Hospital, GenesisCare, 28002 Madrid, Spain; abrahams.ocanto@genesiscare.es (A.O.); daniela.gonsalves@genesiscare.es (D.G.); gloria.guardia@genesiscare.es (G.G.); 2 Department of Radiation Oncology, Vithas La Milagrosa University Hospital, GenesisCare, 28010 Madrid, Spain; 3 Department of Radiation Oncology, Gregorio Marañón General University Hospital, 28007 Madrid, Spain; macarenamaria.teja@salud.madrid.org; 4 Department of Medicine, Faculty of Medicine, Health and Sport Sciences, Universidad Europea de Madrid, 28670 Madrid, Spain; 5 Department of Radiation Oncology, Jiménez Díaz University Hospital, 28040 Madrid, Spain; walter.vasquez@quironsalud.es (W.V.); jluna@fjd.es (J.L.); 6 Department of Radiation Oncology, La Paz University Hospital, 28046 Madrid, Spain; inavarrod@salud.madrid.org (I.N.-D.); rosamaria.morera@salud.madrid.org (R.M.); 7 Department of Radiation Oncology, Clínica Universidad de Navarra, 31008 Pamplona, Spain; mmorenoji@unav.es (M.M.-J.); ndhernandez@unav.es (N.H.); iazinovicga@unav.es (I.A.); 8 Department of Radiation Oncology, National Cancer Institute (INCAN), Mexico City 14080, Mexico; dulcemaurias@gmail.com; 9 Department of Radiation Oncology, Santa Creu i Sant Pau Hospital, 08041 Barcelona, Spain; scarlet.crespo@salutsantjoan.cat; 10 Department of Radiation Oncology, 12 de Octubre University Hospital, 28041 Madrid, Spain; mariana.temes@salud.madrid.org; 11 Department of Radiation Oncology, Amsterdam University Medical Centers, Vrije Universiteit Amsterdam, Cancer Center Amsterdam, 1117 Amsterdam, The Netherlands; s.senan@amsterdamumc.nl; 12 Department of Radiation Oncology, University of California, Los Angeles, CA 90095, USA; dmoghanaki@mednet.ucla.edu

**Keywords:** lung cancer, adaptive radiotherapy, image guidance, magnetic-resonance linear-accelerator

## Abstract

**Simple Summary:**

Magnetic resonance-guided radiotherapy (MRgRT) using MR-linac technology is transforming the treatment of lung cancer by providing superior soft-tissue visualization, real-time assessment of tumor motion and facilitating daily treatment plan adaptation for anatomical changes. These capabilities address key challenges in lung radiotherapy, including managing respiratory motion and permitting safer treatment of tumors in proximity to critical organs. This review summarizes the current evidence and clinical applications of MR-guided adaptive radiotherapy in lung cancer, covering its technical principles, adaptive workflows, patient selection, and motion management strategies. It also discusses the integration of MRgRT into routine clinical practice and explores future developments, such as personalized dose adaptation and the incorporation of functional imaging. MR-linac-based adaptive radiotherapy has the potential to further improve treatment precision and support more individualized care for patients with lung cancer.

**Abstract:**

Magnetic resonance-guided radiotherapy (MRgRT) delivered with MR-linac technology represents a major advance in lung cancer radiotherapy, enabling daily online adaptive planning based on high-quality soft-tissue visualization, real-time motion assessment, and anatomy-driven treatment personalization. The challenges in lung cancer treatment with conventional radiotherapy delivery due to respiratory motion, inter and intra-fraction anatomical changes make MRgRT capabilities particularly relevant. This narrative review provides an overview of the current state of the art of MR-guided adaptive radiotherapy in lung cancer, focusing on technical foundations, adaptive workflows, and clinical implementation across different disease stages. Key aspects as patient selection, adaptive decision-making, motion management strategies, and integration into routine clinical practice are discussed. Finally, emerging clinical applications and future directions—including adaptive dose personalization and integration of functional imaging—are outlined, highlighting the potential role of MR-linac–based adaptive radiotherapy as a platform for precision radiotherapy in lung cancer.

## 1. Introduction

Lung cancer is the most frequently diagnosed malignant tumor and the leading cause of cancer-related deaths worldwide [1]. In 2024, approximately 2.6 million new cases and 1.9 million deaths were reported, with estimates that these values will increase in the next years [2]. The two main subtypes of lung cancer include non-small-cell lung cancer (NSCLC) and small-cell lung cancer (SCLC), with radiation therapy as a key component of treatment for both. Radiation therapy techniques include stereotactic body radiotherapy (SBRT) for early-stage NSCLC and SCLC, chemoradiotherapy in locally advanced NSCLC and SCLC, metastasis-directed SBRT for oligometastatic disease, and finally for palliating symptoms [3,4,5].

Radiotherapy for lung cancer presents several challenges. One challenge is respiratory motion, which can cause significant tumor displacement during treatment and compromise the accuracy of the delivered dose, especially for those lesions located in the lower lobes [6]. Tumor motion can be further compounded by anatomical variability during a treatment course due to tumor shrinkage, shifts, or the development of atelectasis. Intrafraction changes may arise not only due to respiratory motion, but also from cardiac action or unrecognized patient motion in the absence of real-time visualization [7,8]. Furthermore, tumor proximity of tumors to critical organs of interest, such as the heart, esophagus, central bronchial tree or spinal cord, limits delivery of curative doses for central and ultra-central tumors [9,10]. All these factors highlight the need for improved real-time image guidance with the option to adapt treatment plans to optimize outcomes.

In this context, the evolution from conventional CT-based image-guided radiotherapy (IGRT) to magnetic resonance-guided adaptive radiotherapy (MRgRT) represents a new pop-up option [11].

In the absence of controlled clinical trials comparing MRgRT with conventional techniques, the benefits of this treatment for lung cancer are theoretical; however, a narrative review is presented providing an update on the current status of MRgRT in lung cancer treatment by summarizing technological foundations, clinical advances, and the unavoidable challenges of its implementation. Available clinical evidence is explored, and the future perspectives of this emerging modality will be discussed.

## 2. Methods

The articles cited in this narrative review were selected through an exhaustive search of the PubMed, Scopus, Cochrane, Google Scholar, and ScienceDirect databases, with no time restrictions. The search was conducted using the following keywords: (Lung cancer) (Non Small Cell Lung Cancer) (Stereotactic radiotherapy) (Reirradiation) (Protontherapy) (CBCT) (SBRT), and (SABR). Only relevant prospective and retrospective studies published in English between 2010 and 2026 were included.

## 3. MR-Guided Adaptive Radiotherapy: Technology Enabling Clinical Change

### 3.1. MR-Linac Systems and Imaging Capabilities

The integration of magnetic resonance imaging (MRI) with linear accelerators (MR-linac) represents a major advance in image-guided radiotherapy, enabling high-resolution soft-tissue visualization alongside real-time adaptive treatment delivery. Two MR-linac platforms are currently in clinical use: Elekta Unity (Elekta AB, Stockholm, Sweden), which combines a 1.5 Tesla MRI scanner with a 7 MV flattening filter-free linear accelerator, and ViewRay MRIdian (ViewRay Systems Inc., Mountain View, CA, USA), which evolved from an initial cobalt-60 configuration to a 0.35 T MRI-guided linac system developed by ViewRay Inc, which received FDA 510(k) clearance in 2012 [12,13].

Elekta Unity was first clinically deployed in 2017, with CE-marked treatments initiated that same year and FDA clearance granted in 2018 [14]. The MRIdian system entered clinical use in 2014 using cobalt-60 sources, followed by FDA approval of the linac-based platform in 2017 [13]. Today, this system uses a split magnet to generate a 0.35 T field compatible with its integrated linear accelerator. For both, MRI offers superior soft-tissue contrast compared with kilovoltage or megavoltage CT imaging of the chest without additional ionizing radiation [15].

A range of MRI sequences have been used in radiotherapy workflows. T2-weighted imaging enables precise gross tumor volume (GTV) and organ-at-risk (OAR) delineation, while balanced steady-state free precession (bSSFP) and TrueFISP sequences support real-time cine MRI acquisition at 4–8 frames per second for motion monitoring [16,17]. Diffusion-weighted imaging (DWI) and dynamic contrast-enhanced (DCE) MRI images are now available that could provide information for biological target definition and early response assessment, and this is largely confined to research settings or offline evaluation rather than routine online adaptive practice [13]. The higher field strength of the 1.5 T Unity allows for access to a broader range of diagnostic-quality sequences, whereas the 0.35 T MRIdian primarily relies on bSSFP imaging to provide high-quality imaging of the thorax. Both are helpful for treatment localization, while the differences in MRI technology impact the contrast-to-noise ratio, temporal resolution, and susceptibility to geometric distortion [13].

**Figure 1:**
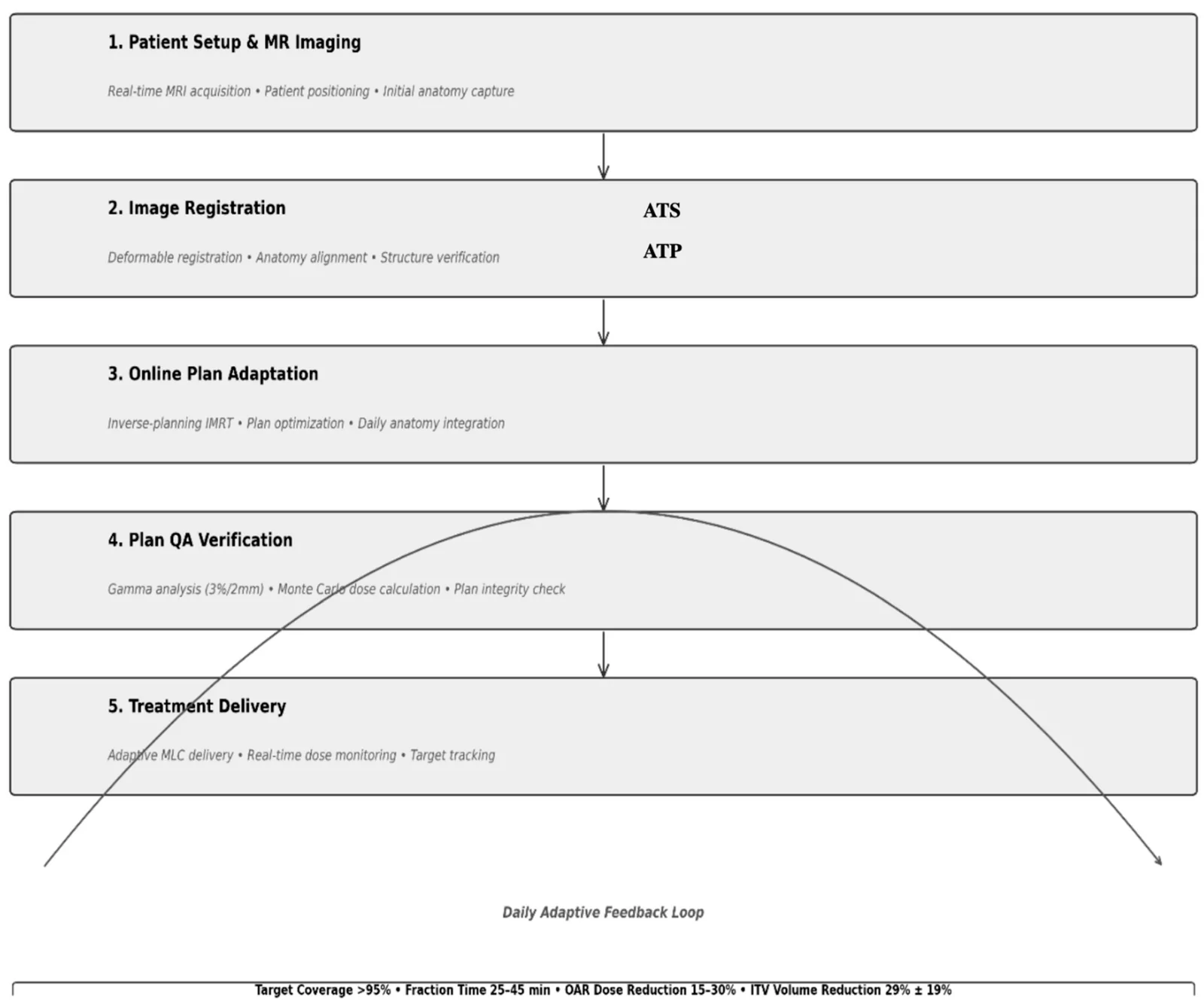
Summary of MRgRT workflow in lung cancer. ATP: Adapt to position. ATS: Adapt to shape.

Online adaptive radiotherapy (oART) is the defining feature of MR-guided workflows. Two principal strategies are employed: adapt to shape (ATS), which involves daily recontouring and full plan re-optimization based on current anatomy, and adapt to position (ATP), in which the original plan is rigidly or deformably registered to the daily anatomy [16]. Daily oART can minimize the risk of excessive doses to critical OARs while enabling target dose escalation, particularly in the presence of significant tumor regression or organ displacement [18]. However, oART workflows are resource-intensive, requiring continuous involvement of a dedicated multi-professional team and can take up to 90 min to deliver each oART-defined treatment (Figure 1) [12]. Active developments in automated segmentation using deep-learning and fast-optimization algorithms are aiming to improve efficiency and clinical scalability of oART [19].

### 3.2. Motion Management and Image Guidance

Respiratory motion represents a major source of uncertainty in thoracic radiotherapy, with lung tumors exhibiting three-dimensional displacements of up to several centimeters, most prominently in the craniocaudal direction [20]. This results in intrafraction variability and interfraction variability due to changes in breathing patterns, tumor regression or progression, and anatomical alterations throughout the treatment course [21].

In conventional cone-beam CT (CBCT)-based image-guided radiotherapy, motion is managed through the internal target volume (ITV) derived from four-dimensional CT (4D-CT), respiratory gating, and deep inspiration breath-hold (DIBH). While effective, these strategies often increase the volume of irradiated normal lung tissue and rely on intermittent imaging with limited soft-tissue contrast, preventing detection and correction of intrafraction motion during beam delivery [21,22].

MRgRT provides a fundamentally different paradigm by enabling continuous real-time tumor visualization. Cine MRI allows beam gating based on direct observations of the tumor position rather than external surrogates or implanted fiducial markers [17]. The MRIdian system employs a gating-on-target approach, in which radiation delivery is automatically interrupted when the tumor moves outside a predefined boundary; this technique was first clinically validated in 2018 [23].

Direct tumor visualization is particularly advantageous in lung cancer and provides a safer alternative to fiducial marker implantation techniques which carry procedural risks. It also avoids a reliance on external respiratory surrogates that may inadequately reflect internal tumor motion, especially in patients with irregular breathing patterns or treatment-induced internal anatomical changes [21]. MR-guided gated SBRT has demonstrated high geometric accuracy, achieving ≥95% GTV coverage with duty cycles ranging from 67% to 87% [24]. This precision enables the use of reduced planning-target-volume (PTV) margins, thereby minimizing irradiation of surrounding healthy tissues [25].

Another key strength of MRgRT lies in the integration of real-time imaging with adaptive planning. Daily MRI acquisition allows for detection of clinically significant interfraction changes, including tumor shrinkage, pleural effusion, or mediastinal shifts, and online replanning corrects their dosimetric impact prior to treatment delivery capabilities not achievable with CBCT-based workflows [15,16]. Comparative studies have demonstrated improved sparing of organs at risk, including the lungs, esophagus, heart, and brachial plexus, particularly in stereotactic treatments for early-stage NSCLC and centrally located tumors [26].

Overall, MRgRT integrates multiple motion management strategies (including gating, breath-hold, and emerging tracking approaches) within a unified adaptive framework, representing a significant evolution beyond conventional image-guided techniques [27]. However, all of these factors point to greater precision, which must be demonstrated in randomized clinical trials to determine the true impact on tumor control.

## 4. Clinical Implementation Across Lung Cancer Scenarios

### 4.1. Early-Stage NSCLC and Stereotactic Body Radiotherapy

SBRT has become the standard of care for medically inoperable early-stage NSCLC, achieving local control (LC) rates exceeding 90% for peripheral tumors [28,29]. However, the risk–benefit profile varies depending on tumor location. MRgRT may be particularly promising for treatment in higher-risk anatomical settings where conventional SBRT has limitations [30].

#### 4.1.1. Peripheral Tumors

For peripheral tumors located >2 cm from the proximal bronchial tree (PBT), SBRT with biologically effective doses (BED_10_) ≥100 Gy yields excellent LC (>95%) and low rates (<10%) of grade ≥ 3 adverse events, as reported in the phase II RTOG 0915 and phase III CHISEL trials [31,32,33]. In this setting, MR-guided adaptation offers limited additional benefits, as anatomical variations are usually small, and 4D-CT-based motion management is sufficient [30,33]. Nevertheless, MRgRT may offer advantages in selected tumors with respiratory motion exceeding 1 cm, re-irradiation, prior thoracic surgery, or proximity to the chest wall. It can also optimize visualization of tumors near the diaphragm, where simulation images are often associated with substantial artifact and visual uncertainties. In these situations, daily MRI-based guidance with repeated breath-hold motion mitigation strategies allows for safer margin reduction, lowering the lung V_20_ and chest-wall dose to minimize the risk of treatment-related adverse events [30,34].

#### 4.1.2. Central and Ultra-Central Tumors

**Figure 2:**
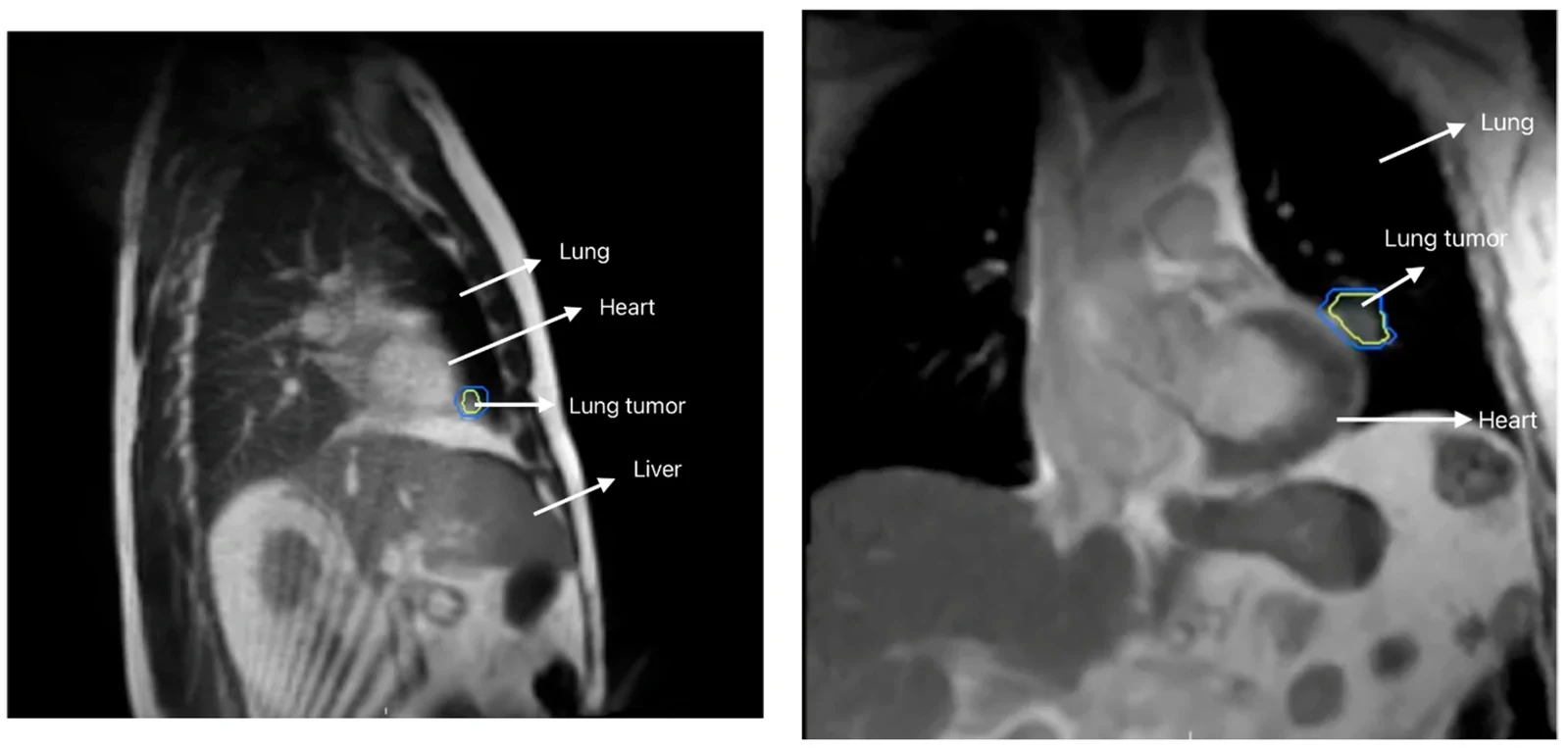
NSCLC pericardiac (ultra-central lesion) treated with gating and PTV of 2 mm. Dose of 50 Gy in 5 fractions. No adverse events > G3 were reported.

MRgRT for thoracic targets is most relevant for tumors near critical mediastinal structures. Central tumors (within 2 cm of the PBT) and ultra-central lesions (adjacent to PBT, trachea, esophagus, or great vessels) have significantly higher side-effect risks with high-dose SBRT treatments (Figure 2). Therefore, MRI-guided treatments in this setting offer advantages due to their ability to distinguish between soft tissues and, in theory, would be an alternative to conventional treatment; however, there is a lack of randomized studies to support this indication. The HILUS trial, delivering 56 Gy/8 fractions to ultra-central lung tumors, reported a 34% rate of grade ≥ 3 adverse events, including 15% fatal bronchopulmonary hemorrhage, largely attributed to an excessive dose to the tracheobronchial wall [35,36]. RTOG 0813 identified 12 Gy per fraction (60 Gy/5 fractions) as the maximum tolerated dose for central tumors, achieving 88% 2-year LC including 3.3% fatal complications [37]. The SUNSET phase I trial subsequently defined 60 Gy/8 fractions with a hotspot ≤ 120% as a safe regimen for ultra-central tumors, with 7% grade ≥ 3 adverse effects at a median follow-up of 36.5 months [38]. Theoretically, MRgRT may reduce adverse effects in central and ultra-central tumors due to improved image guidance, and accordingly, may allow for a potential dose escalation; however, clinical trial results are still needed to translate this idea into actual clinical practice.

#### 4.1.3. Side-Effect Mitigation Through Online Adaptation

Clinical evidence has demonstrated the ability of real-time MR-guided adaptation to improve dosimetry in high-risk lung SBRT plans. Finazzi et al. reported the utility of plan adaptation that was performed in 68% of fractions for central and ultra-central tumors treated on the MRIdian system, achieving 96% 1-year LC with <5% severe adverse events [39]. The SMART trial (stereotactic MR-guided adaptive radiotherapy) for ultra-central lesions showed reductions of 5.7 Gy in PBT D_max_ that also improved target coverage by 7.3% [40]. Merckel et al. described tumor shrinkage of up to 58% during treatment using MR-linac (1.5 T Unity), with no severe acute adverse events [41]. These results support MRgRT as a preferred approach for central and ultra-central tumors. Ongoing trials (MAGELLAN, STAR-LUNG, and STRICT-LUNG) are further evaluating its safety and efficacy [42,43]. More studies are necessary in this scenario to translate theoretical dosimetric benefits into local control, quality of life, and, above all, impacts on survival and in adverse events. For now we do not have any preference for one technique relative to another. The main studies are summarized in Table 1.

### 4.2. Locally Advanced Lung Cancer

Locally advanced non-small-cell lung cancer is another scenario in which MRgRT may be beneficial. Although concurrent chemoradiotherapy (CRT) followed by durvalumab is the standard of care [44], locoregional failure persists in 30–40% of patients, and dose escalation attempts have failed due to overdose to critical structures [44,45,46]. MRgRT may help overcome this limitation by adapting the radiation plan to tumor and anatomical changes throughout treatment and reducing doses in OARs for minimal PTV [47,48].

#### 4.2.1. Interfraction and Intrafraction Anatomical Changes

Locally advanced NSCLC is one of the most anatomically dynamic disease sites in radiation oncology. Kwint et al. analyzed 1793 CBCTs in 177 patients and identified clinically significant intrathoracic changes, including tumor regression, atelectasis, pleural effusion, and mediastinal shift, in approximately 70% of patients during the treatment course [48]. Møller et al. reported a mean GTV reduction of 38% across 233 patients undergoing CRT, which, without replanning, resulted in overdose to surrounding critical structures and undercoverage of the target [49]. Intrafraction motion also adds complexity: respiratory motion, baseline drift, and cardiac movement create significant uncertainties in dose delivery [20]. MR-linacs address both interfraction and intrafraction changes using daily MRI for plan adaptation and real-time cine MRI for beam gating, allowing for direct tumor tracking without ionizing radiation [46,47]. Early data suggest that MR-guided daily adaptation can improve PTV coverage from 33% to 98% compared to non-adapted plans [42], Theoretically, in the absence of studies, this could help with local disease control with minimal adverse events. However, in the absence of controlled studies, doubts remain as to whether a reduction in treatment volumes may affect local control, as well as whether the area of microscopic involvement within the original volume is well defined and whether there is variation in daily contouring if it is not performed by the same physician. Another variable is the reduction in margins, and we do not know whether this influences cancer control. These uncertainties continue to make CB-CT-guided treatment (which has been extensively validated in clinical studies) the first-line treatment option for lung cancer when radiation therapy is required.

#### 4.2.2. Potential Role of Adaptive Strategies in Conventionally Fractionated RT

Two adaptive paradigms are emerging for locally advanced NSCLC, both supported by MRgRT. First, critical structure–sparing adaptation maintains the prescribed dose (60–66 Gy) while reducing the target to the residual tumor volume, thereby lowering the dose to the lung, heart, and esophagus. The LARTIA prospective trial demonstrated that weekly CT-guided replanning achieved 83% 2-year LC while significantly reducing the lung V_20_ and esophageal dose compared to historical controls [50]. Second, isotoxic dose escalation uses the spared dose to increase radiation to the residual tumor. Planning studies suggest that doses of 75–80 Gy can be delivered within critical structure constraints, unlike in RTOG 0617 where dose escalation was performed without adaptive planning [45,51]. The PUMA phase I trial will evaluate MRgRT in locally advanced NSCLC, with primary endpoints of feasibility (successful adaptation in ≥80% of fractions) and treatment efficiency (on-table time < 90 min) and secondary endpoints including adverse effects, LC, and overall survival (OS) [46]. The results from PUMA and related ongoing initiatives (MAGELLAN, ARTIA-Lung, and STARLUNG) will be critical in determining whether MRgRT can finally translate its dosimetric superiority into improved clinical outcomes for this challenging patient population [43,52]. Key studies on adaptive radiotherapy in locally advanced NSCLC are summarized in Table 2.

### 4.3. Oligometastatic and Metastatic Lung Disease

#### 4.3.1. Adaptive SBRT for Pulmonary Metastases

Evidence regarding MRgRT for pulmonary metastases is encouraging but remains limited and continues to evolve. Finazzi et al. reported favorable LC outcomes with acceptable side-effect profiles in patients with pulmonary metastases, supporting the clinical utility of daily adaptation to maintain target coverage and organ-at-risk constraints [39]. The multicenter phase II SMART ONE trial evaluated single-fraction SBRT delivered on an MR-linac for patients with lung lesions, providing prospective evidence of the feasibility and safety of extreme hypofractionation in this setting [53]. Given that the potential benefits of MRgRT may be greatest in complex anatomical situations, the prospective MAGELLAN study for ultra-central thoracic tumors incorporates MR-guided adaptation and respiratory gating strategies to mitigate treatment-related complications, with definitive results awaited [43]. Similarly, a systematic review of MR-guided radiotherapy for oligometastatic disease concluded that superior soft-tissue visualization and real-time adaptation may improve treatment precision while reducing radiation exposure to organs at risk; however, the authors highlighted important methodological limitations and the need for comparative prospective evidence [54]. In parallel, existing SBRT guidelines for pulmonary oligometastasis have demonstrated favorable outcomes and provide an important benchmark against which the incremental value of MRgRT can be assessed [55]. However, in the context of multimodal treatment, MRgRT in patients with oligometastases is beneficial for preserving healthy tissue and preventing the need for further radiation therapy, using ablative doses. Further studies are needed in this setting to support this indication.

#### 4.3.2. Motion-Adaptive Dose Escalation Strategies

Motion management is critical when considering dose escalation in lung SBRT. In this context, MRgRT combined with online adaptive planning may offer significant advantages by reducing intrafraction uncertainties, enabling smaller treatment margins, and maintaining dosimetric constraints for OARs. The ESTRO ACROP recommendations support the implementation of respiratory motion management as a standard approach to mitigate breathing-induced target motion, particularly in the setting of high-precision SBRT [56]. In clinical practice, studies evaluating MR-guided SBRT have demonstrated the potential benefits of real-time motion monitoring. Ehrbar et al. showed that cine MRI-guided beam gating substantially reduced residual target motion and prevented target under-dosage compared with treatments delivered without gating, supporting the role of real-time monitoring when pursuing BED escalation without increasing PTV margins [57]. For peripheral lung tumors, Finazzi et al. reported that a SMART approach incorporating breath-hold and respiratory gating enabled smaller PTV margins compared with conventional ITV-based strategies, thereby creating favorable conditions for safe dose escalation [58]. In more challenging clinical scenarios, such as ultra-central tumors or re-irradiation cases, radiation doses are often limited by the proximity of OARs. In these situations, the combination of advanced respiratory motion management and MRgRT may facilitate safer delivery of ablative dose levels while maintaining OAR constraints [58]. Overall, the available evidence indicates that MRgRT, combined with real-time respiratory motion management, provides a promising platform for dose escalation in lung SBRT by enhancing treatment precision and protecting surrounding normal tissues through patient-specific adaptive planning.

### 4.4. Re-Irradiation in Lung Cancer

#### Rationale for MR-Guided Adaptation

Evidence supporting lung re-irradiation using MRgRT/SMART remains limited, with most available data originating from retrospective series. The overall safety framework is derived from re-irradiation not using MRgRT. The American Radium Society (ARS) Appropriate Use Criteria, based on a systematic review of 52 studies (only three of which were prospective), recommend the use of highly conformal techniques, including IMRT, VMAT, and SBRT, for patients undergoing curative-intent thoracic re-irradiation. These recommendations emphasize the importance of accounting for cumulative radiation doses from prior treatments and respecting EQD2-based organ-at-risk constraints to minimize severe side effects. Suggested dose limits include esophageal V60 < 40% and Dmax < 100 Gy, lung V20 < 40%, proximal bronchial tree/trachea Dmax < 110 Gy, aorta and major vessel Dmax < 120 Gy, and spinal cord Dmax < 57 Gy. The guidelines also highlight the increased risk associated with central lesions and overlapping treatment fields [59,60]. In this context, one of the few reports evaluating the use of MRgRT in lung re-irradiation comes from the series published by Finazzi et al. Patients were treated using daily online adaptation combined with MR-guided breath-hold and respiratory gating, and the cohort included 17 re-irradiation cases. In the overall study population, the authors reported a 12-month local control rate of 95.6% and a grade ≥ 3 adverse event rate of 8%, with no grade 4–5 adverse events. Although local failures included some of the re-irradiated lesions, these findings suggest that MR-guided adaptive SBRT is a feasible treatment approach with an acceptable safety profile despite the challenges posed by previously irradiated tissues and reduced normal tissue tolerance [39]. From a technical perspective, reviews of MRgRT have proposed that improved soft-tissue visualization, real-time intrafraction monitoring, and online adaptive planning may be particularly advantageous in the re-irradiation setting, where even minor anatomical variations can result in clinically relevant overdosing of critical structures. Nevertheless, the current data are largely hypothesis generating, and this limitation should be acknowledged when interpreting the potential benefits of MRgRT in this context [59,60]. Based on the currently available evidence, MRgRT appears to be a feasible option for selected patients undergoing lung re-irradiation. When evaluated on a case-by-case basis, this approach may offer potential dosimetric and clinical advantages while maintaining an acceptable safety profile when the appropriate technology is available. However, its role remains to be defined through dedicated prospective studies, as well as through the standardization of cumulative dose assessment methodologies and the systematic reporting of late adverse events [58,59,60].

## 5. Dosimetric and Biological Impact of MR-Guided Adaptation

### 5.1. Margin Reduction and Dosimetric Benefits

The integration of MRgRT in lung cancer has proven particularly advantageous for SBRT delivery, where this technology facilitates real-time tumor monitoring and seamless online adaptive planning [61]. In a lung-target subgroup of a multi-institutional analysis, a consistent improvement in GTV coverage was observed. The most significant median improvements were found in the GTV near-minimum dose (D98%), which increased by 3.9% (*p* < 0.001) [62]. Similarly, a retrospective analysis of 36 patients (comprising 294 fractions) demonstrated that daily adaptation improved PTV coverage from a predicted 92.7 ± 5.4% in re-optimized plans (*p* < 0.001). This improvement was accompanied by a significant reduction in the maximum point dose (Dmax) to various OARs, including the PBT (5%), heart (4.8%), and brachial plexus (4.7%) [63]. A direct comparison of MRgRT with conventional CT-based planning reveals quantifiable dosimetric advantages across multiple clinical endpoints. In a prospective study comparing MR-based versus CT-based tumor delineation for locally advanced NSCLC, MR-based delineation achieved significantly smaller planning target volumes (186.1 vs. 315.3 cm^3^, *p* < 0.001). This volumetric reduction correlated with a lower incidence of grade ≥G2 pneumonitis (10% vs. 24.4%, *p* = 0.001) and ≥G3 esophagitis (2.2% vs. 15.6%, *p* < 0.001) [64]. The most significant clinical benefit of MRgRT is the ability to substantially reduce PTV margins while maintaining robust target coverage. In a retrospective study of apical lung lesions, cine-MRI analysis demonstrated GTV motion quantification of <2.2 mm in both anteroposterior and craniocaudal directions. The study noted that a 3 mm margin would be sufficient to maintain GTV coverage while reducing the chest-wall Dmax by 2.5 Gy and the tracheobronchial Dmax by 3.0 Gy [65]. Furthermore, real-time tracking and predictive gating allow for margin reductions lower than the standard 5 mm used clinically. This is achievable even when utilizing surrogate structure tracking in cases where the primary targets are difficult to visualize using conventional imaging modalities [66].

### 5.2. Target Delineation and Contouring Under MR Guidance

The fundamental advantage of MRI in radiotherapy treatment planning is centered on three critical pillars: superior soft-tissue contrast, volumetric precision, and computational efficiency. Unlike conventional CT, MRI provides high-resolution contrast to distinguish malignant lesions from adjacent confounding pathologies, such as lung atelectasis, thereby facilitating more accurate contouring and the adoption of smaller target volumes [67]. Furthermore, the integration of deep-learning-based auto-segmentation and contour propagation methods has significantly streamlined the clinical workflow [68]. These AI-driven advancements not only improve overall planning efficiency but also critically reduce the inter-observer variability traditionally associated with the manual delineation of tumors and OARs [69,70].

### 5.3. Critical Organs at Risk and Adverse-Event Mitigation

The clinical significance of MRgRT becomes most apparent in the context of organ-at-risk sparing and the reduction in radiation-induced adverse effects, such as pneumonitis risk reduction of grade 2 pneumonitis of 59% (*p* = 0.001) esophagitis risk reduction of grade 3 esophagitis of 86% (*p* = 0.001) and cardiac complications [70] (Table 3).

### 5.4. Radiobiological Implications of Daily Online Adaptation

MRgRT provides daily plan recalculation to “recalculate” the cumulative dose in OARs based on the anatomy of the day. Unlike static planning, where damage accumulates geometrically, MRgRT ensures each fraction is physiologically calibrated to the current target and OAR configuration [76]. Beyond single-fraction gains, accumulation using deformable image registration reveals that MRgRT provides a compounding benefit [77]. While specific lung-dose accumulation studies are still limited, data extrapolated from rectal cancer models demonstrate that adaptation prevents unintended “hotspots” (e.g., PTV D_2_%) and OAR overdosing caused by inter-fraction anatomical shifts [66,78].

### 5.5. Functional MRI, Radiomics, and Artificial Intelligence

Beyond conventional morphological sequences, advanced functional MRI provides quantitative biomarkers that are revolutionizing adverse-event prediction and response assessment. At this time, it is not available for clinical use, although the results seem promising. A critical advancement is the use of 2D-cine MRI and Hyperpolarized Xenon-129 (^129^Xe) MRI for ventilation-based lung sparing. While traditional dosimetric parameters (MLD, V20, and V5) often fail to distinguish patients at risk for radiation pneumonitis (RP), functional MRI-derived ventilation maps have demonstrated a high predictive value (AUC = 0.84, *p* < 0.05) [79]. Specifically, ^129^Xe MRI allows for a paradigm shift by imaging aerated lung regions and gas exchange across the alveolar-capillary membrane. In NSCLC patients, function-based planning using (^129^Xe) MRI significantly reduced doses to well-ventilated lungs—decreasing V20% from 12.5% to 11.0% (*p* < 0.05), which translates to a clinically substantial NTCP reduction equivalent to a 3.5–8 Gy dose-response improvement [64]. Complementing this functional data, radiomics extracts high-dimensional features to characterize tumor heterogeneity. Research confirms the robustness of these features against inter-observer variability, with semi-automated datasets achieving a median intraclass correlation coefficient of 0.93, ensuring that first-order and texture-based features remain reliable for clinical decision-making [70]. Finally, Artificial Intelligence (AI) serves as the integrative engine for these modalities. AI-powered auto-segmentation and deep-learning frameworks have optimized the adaptive workflow, particularly in selecting the optimal strategy, ATP versus ATS. Recent models have achieved an AUC of 0.861 for strategy selection in just 2.5 min, a five-fold efficiency increase over traditional methods [26]. The convergence of functional MRI, radiomics, and AI is expanding the precision of MRgRT, moving us toward a highly personalized, biologically guided radiotherapy of the future [61].

## 6. Practical Challenges and Comparison with Other Technologies

### 6.1. Workflow Complexity, Treatment Time, and Resource Requirements

MRgRT for lung cancer allows for proper demarcation with tumor delineation accuracy and enhanced sparing of OARs. However, these treatments are associated with a significant increase in workflow complexity, technical requirements, and treatment duration, posing challenges for its routine clinical implementation. The workflow of lung MRgRT involves multiple sequential steps performed while the patient remains on the treatment couch. First, a daily magnetic resonance image is acquired. This image is then registered to the planning dataset and subsequently reviewed and manually adjusted by the radiation oncologist. Following this process, treatment plan re-optimization is performed, followed by quality assurance verification. Finally, treatment delivery is carried out using real-time cine-MR imaging and respiratory gating techniques [26]. The entire process results in considerably longer treatment times (typically ranging from 45 to 90 min) and greater technical demands compared with conventional radiotherapy. From a technical perspective, lung MRgRT faces several intrinsic limitations related to magnetic resonance imaging of pulmonary tissue, including low proton density and susceptibility artifacts, which may compromise image quality and hinder visualization of small lesions. During treatment planning, the electron return effect, resulting from the interaction between the magnetic field and secondary electrons, may generate dose heterogeneities at air–tissue interfaces [11,26]. During treatment delivery, factors such as respiratory gating reduce the effective irradiation time (duty cycle), particularly in highly mobile tumors, thereby contributing to prolonged treatment sessions [80]. Nevertheless, institutional experience, the use of dedicated MRI acquisition sequences, auto-contouring tools, and patient familiarization with techniques such as breath-hold can progressively improve workflow efficiency and reduce treatment times [81]. It should be noted that when beginning MRgRT treatments, there is a learning curve, which means that each treatment takes longer; this duration gradually shortens with experience but typically lasts about an hour. In no case has the MRgRT treatment workflow been shown to impact disease control; however, it does make the treatment less comfortable for the patient due to the long time spent on the machine, compared to conventional linear accelerators.

### 6.2. Cost-Effectiveness and Access Considerations

Currently, no studies have specifically evaluated the cost-effectiveness of MRgRT in lung cancer. However, data extrapolated from other thoracoabdominal malignancies affected by respiratory motion, such as hepatocellular carcinoma, suggest an approximately 18% increase in direct costs compared with CT-guided SBRT [82]. These analyses demonstrate that the additional cost associated with MRgRT is primarily driven by increased machine occupancy time and specialized personnel requirements rather than consumable resources. Consequently, these costs may be reduced through workflow optimization. Furthermore, such analyses do not account for potential cost reductions achievable through treatment strategies such as single-fraction radiotherapy. Importantly, potential clinical benefits (particularly the reduction in severe treatment-related complications in centrally located lung tumors) should also be considered, as they may partially offset the higher initial investment. In this context, dedicated prospective studies are needed to better define the true value of this technology. Current access to MRgRT is largely confined to centers in high-income countries. For much of the world, the primary challenge continues to be ensuring access to high-quality conventional radiotherapy. As a result, from a global oncology equity perspective, widespread implementation of MRgRT remains a distant objective.

### 6.3. MR-Guided Adaptive Radiotherapy Versus CBCT-Based Adaptive Approaches

MRgRT and CBCT-based adaptive radiotherapy differ substantially in both technical capabilities and clinical applicability. CBCT-based adaptive radiotherapy currently represents the most widely adopted adaptive platform due to its broad availability and operational efficiency, including relatively short treatment times. Theoretically, CB-CT positioning images are inferior to MRI in terms of poor soft-tissue contrast inherent to CBCT imaging, which restricts online adaptation capabilities reducing responsiveness to anatomical changes occurring during treatment [82]. But as in other settings, there are no comparative studies demonstrating the superiority of one technique over another. However, the ability to generate low-voltage, AI-enhanced images results in a reduction in treatment time for CB-CT devices compared to MRgRT, which translates into a theoretical advantage for these systems (Table 4).

### 6.4. MR-Guided Adaptive Radiotherapy Versus Proton Therapy

The principal advantage of proton therapy is dosimetric. The Bragg peak, defined as the loss of energy by ionizing radiation as it travels through matter (typical of heavy particles), allows for highly conformal dose distributions with a sharp distal dose fall-off, potentially providing superior sparing of organs at risk (heart, lungs, etc.) compared with photon-based treatments. However, this same characteristic represents its main vulnerability in lung cancer, as interfraction anatomical changes and variations in tissue density can alter the beam range, increasing the risk of inadequate target coverage or unintended overdosing of adjacent normal structures. Respiratory motion management introduces an additional challenge, since interplay effects may limit the effectiveness of conventional motion-compensation strategies, and online adaptive proton therapy has not yet been widely implemented [83,84]. As with other scenarios, we lack comparative studies; it is necessary to determine whether the variability in interaction that can be adjusted in MRgRT in a more visually accurate manner than with protons actually has an impact on disease control, or whether, on the contrary, the reduced toxicity to adjacent tissues associated with proton therapy paves the way for dose escalation and prepares the surrounding tissue for subsequent courses of radiation therapy in long-term survivors (Table 4).

## 7. Future Directions and Research Priorities

### 7.1. Adaptive Dose Personalization and Biologically Guided Radiotherapy

Current MRgRT workflows adapt treatment geometry daily; the next frontier is to explore and validate biological dose personalization. At 1.5 T, multiparametric sequences (DWI, DCE-MRI, and BOLD imaging) characterize tumor hypoxia, cellularity, and perfusion, enabling voxel-level dose redistribution toward radioresistant subvolumes [85]. Changes in the apparent diffusion coefficient (ADC) during chemoradiotherapy for NSCLC serve as early biomarkers of tumor response, identifying subvolumes that may benefit from dose escalation [86]. Margin reduction from 7 mm to 3 mm PTV margins in locally advanced NSCLC facilitates isotoxic dose escalation while respecting normal tissue constraints [87]. Delta-radiomics on longitudinal on-treatment MR images predicts which patients benefit most from adaptive replanning, enabling pre-treatment stratification and mid-course dose modification [88]. As predictive neural networks that forecast spatial tumor evolution integrate into clinical workflows [89], biologically informed on-table adaptation will transform MR-linac into a closed-loop, response-adaptive platform.

### 7.2. Integration of MR-Guided Radiotherapy with Immunotherapy or Targeted Therapy

The combination of radiotherapy and immunotherapy has generated substantial enthusiasm, fueled by the abscopal effect (localized irradiation inducing systemic antitumor immune responses at distant sites) [90]. MRgRT optimizes this combination: precise delivery enables hypofractionated regimens that maximize immunogenic cell death, while sparing thoracic lymph node chains minimizes radiation-induced immunosuppression, a predictor of impaired checkpoint inhibitor benefits [90]. A landmark phase 2 trial showed that primary lung tumor SBRT followed by concurrent mediastinal chemoradiotherapy and adjuvant durvalumab was feasible and efficacious in locally advanced NSCLC [90]. For EGFR- or ALK-driven NSCLC, MRgRT daily pulmonary dose optimization may open a therapeutic window currently deemed unsafe with conventional radiotherapy [90].

### 7.3. Ongoing and Planned Clinical Trials

The evidence base for MRgRT in lung cancer is rapidly expanding. The international MOMENTUM registry collects clinical, dosimetric, and imaging data across all MR-linac platforms, establishing foundational real-world evidence for MRgRT benefits [91]. The MR-Linac Consortium’s dedicated lung tumor site group is developing standardized functional MRI protocols, validating auto-segmentation algorithms, and designing comparative trials [92]. No randomized trials have yet directly compared MRgRT with CBCT-based adaptive therapy or standard LINAC-SBRT in lung cancer. Planned priorities include MRgRT-based CRT trials in locally advanced NSCLC with the integration of immune checkpoint inhibitors (ICIs), dose-escalation platforms for central tumors, and phase I/II studies combining MR-guided SBRT with novel immunotherapeutic agents [92,93]. Table 5 summarizes the most relevant ongoing and recently published studies.

### 7.4. Towards Personalized Precision Radiotherapy in Lung Cancer

MRgRT is advancing from a technology demonstration to an integrated precision medicine platform. The simultaneous imaging and treatment capabilities of MR-linac systems represent a new paradigm for precision adaptive radiotherapy [92]. The overarching vision is a closed-loop adaptive system: baseline multiparametric MRI defines radioresistant subvolumes, daily imaging drives geometric adaptation, and weekly functional imaging adjusts dose prescriptions according to biological response [85,93]. Patient-specific deep-learning algorithms for lung-tumor auto-segmentation on MRI will enable automated adaptive workflows and expand MRgRT access beyond specialized centers [95]. Realizing this vision requires standardized thoracic MR protocols across institutions, prospective validation of imaging biomarkers (ADC and perfusion) against clinical endpoints, and the integration of multi-omics data—genomics, liquid biopsy, and circulating tumor DNA with on-treatment imaging [62,93]. Given lung cancer’s biological heterogeneity, respiratory motion complexity, and systemic immune interactions, multicenter randomized studies are essential to validate adaptive and combination strategies and establish personalized precision MRgRT as the standard of care [91,94].

## 8. Limitations of MRgRT Use

Despite its potential clinical and dosimetric advantages, the implementation of MRgRT in lung cancer remains challenging. Key barriers include the inherent complexity of the workflow, which involves MRI-based simulation, image registration, daily contour review and/or correction, online replanning, and real-time respiratory motion management, resulting in longer treatment times than conventional linac-based radiotherapy. These requirements also translate into substantial resource demands, including specialized MR-linac infrastructure; dedicated multidisciplinary teams; and trained radiation oncologists, physicists, radiographers, and dosimetrists. In addition, variability in target and organ-at-risk delineation remains an important source of uncertainty, particularly in the presence of anatomical changes, respiratory motion, and the lower familiarity of some clinicians with MRI-based anatomy compared with CT-based planning. The learning curve associated with MRgRT, encompassing MRI interpretation, contouring, adaptive planning, and workflow integration, may further influence treatment efficiency and reproducibility across institutions. Consequently, the adoption of MRgRT requires not only access to the technology but also appropriate training, standardized workflows, and institutional experience. These practical limitations should be considered when evaluating the potential benefits of MRgRT, particularly given that its clinical superiority over contemporary conventional or CT-guided adaptive radiotherapy has yet to be demonstrated in comparative trials.

## 9. Conclusions

MRgRT should not currently be considered a universal replacement for conventional image-guided radiotherapy in lung cancer, as the available evidence remains predominantly based on dosimetric analyses, feasibility studies, and single-arm clinical series. Its incremental value appears to be greatest in anatomically challenging situations in which conventional approaches are limited by respiratory motion, proximity to critical organs, or cumulative dose constraints. In peripheral tumors, where conventional SBRT already provides excellent local control with low toxicity, the additional benefit of MRgRT is likely to be limited, although selected cases with substantial respiratory motion, difficult visualization, prior surgery, or re-irradiation may derive greater benefits. In contrast, central and particularly ultra-central tumors represent one of the most compelling potential applications, as real-time motion monitoring and daily online adaptation may improve target coverage while reducing doses to critical mediastinal structures and potentially widening the therapeutic window. Similarly, lung re-irradiation may represent another particularly relevant scenario, given the limited tolerance of previously irradiated tissues and the potential value of anatomy-of-the-day adaptation, although current evidence remains largely retrospective and hypothesis-generating. In locally advanced disease and pulmonary oligometastatic disease, MRgRT offers a strong dosimetric and technical rationale for adaptive treatment, but whether these advantages translate into improved tumor control, toxicity, quality of life, or survival remains uncertain. Overall, the current evidence supports considering MRgRT selectively rather than routinely, with its greatest potential likely concentrated in re-irradiation and central/ultra-central tumors, while prospective comparative and randomized studies are still required to define which patients derive a clinically meaningful benefit and to establish its role relative to contemporary CT-guided adaptive and conventional radiotherapy approaches.

## Figures and Tables

**Table 1:** Key studies of SBRT and MR-guided adaptive radiotherapy in early-stage NSCLC.

Study	Design	N	Location	Regimen	2 Y LC	Side Effects ≥ G3	Key Relevance for MRgRT
RTOG 0236 [32]	Phase II	55	Peripheral	54 Gy/3 fx	98% (3 y)	16%	Benchmark for peripheral SBRT
RTOG 0813 [37]	Phase I/II	71	Central	50–60 Gy/5 fx	88% (2 y)	7% G3–4	MTD = 12 Gy/fx; highlights need dose precision
CHISEL [33]	Phase III	101	Peripheral	54 Gy/3 fx vs. 66 Gy/33 fx	Superior SBRT	Similar	Standard for peripheral tumors; context for MRgRT benefits
HILUS [35]	Phase II	65	Ultra-central	56 Gy/8 fx	83% (2 y)	34% (15% G5)	High fatal hemorrhage justifying MRgRT approach
Expanded HILUS [36]	Pooled analysis	230	Central and Ultra-central	56 Gy/8 fx	84% (2 y)	13% G5	Bronchial Dmax and compression = G5 predictors; MRgRT targets these
SUNSET [38]	Phase I	30	Ultra-central	60 Gy/8 fx (Dmax ≤ 120%)	82% (2 y)	7% (1 G5)	Defines safe non-MR regimen; MRgRT may further improve window
Finazzi et al. [39]	Prospective	50	Central and Ultra-central	SMART (MRIdian)	96% (1 y)	4%	Adaptation in 68% of fx; key MRgRT feasibility evidence
Merckel et al. [41]	Prospective	10	Ultra-central	60 Gy/8–12 fx (1.5 T MR-linac)	N/R	0% G ≥ 3	First Unity MR-linac experience for UC tumors

fx = fractions; G = grade; Gy = Gray; LC = local control; MTD = maximum tolerated dose; N/R = not reported; UC = ultra-central; SMART = stereotactic MR-guided adaptive radiotherapy.

**Table 2:** Key studies on adaptive radiotherapy in locally advanced NSCLC, with relevance to MR-guided approaches.

Study	Design	N	Modality	Main Finding	Relevance for MRgRT
RTOG 0617 [45]	Phase III	464	3D-CRT/IMRT 60 vs. 74 Gy	High-dose arm led to worse OS; cardiac dose is key driver	Motivates adaptive dose escalation without OAR overdose
PACIFIC [44]	Phase III	709	CRT + durvalumab	Improved PFS and OS with consolidation immunotherapy	Defines standard-of-care context for RT optimization
Kwint et al. [48]	Retrospective	177	CBCT analysis	Major anatomical changes in ~70% during RT course	Quantifies magnitude of problem that MRgRT must address
Møller et al. [49]	Prospective	233	CBCT-guided ART	Mean GTV reduction of 38%; ART preserved coverage and reduced lung dose	Establishes ART benefit; MRgRT enables daily implementation
LARTIA [50]	Phase II	217	Weekly CT-based ART	2 y LC achieved 83% and reduced lung V20 and esophageal dose	Key clinical evidence for adaptive OAR-sparing RT
Guckenberger et al. [51]	In silico	13	CT-based ART	Escalation to 75–80 Gy is feasible with adaptive replanning	Planning rationale for isotoxic escalation with MRgRT
PUMA [46]	Phase I	30	MR-linac	Evaluates feasibility, timing, and AE of MRgRT in LA-NSCLC	First prospective MRgRT trial in LA-NSCLC

CRT = chemoradiotherapy; ART = adaptive radiotherapy; GTV = gross tumor volume; LC = local control; OS = overall survival; OAR = organ at risk; PFS = progression-free survival; RT = radiotherapy.

**Table 3:** OAR structures and advantages in treatments with MRgRT.

OAR Structure	Study	Dose Reduction with MRgRT	Side-Effect Grade Reduction	MRI Advantage	Mechanism
Esophagus	La Rosa et al., 2023 [71]	11.34% mean reduction (cumulative plan)	Grade ≥2 reduced 2–8% absolute	Soft tissue enhanced, and reduced over contour	Daily soft-tissue re-planning
Airway	La Rosa et al., 2023 [71]	5.62% mean dose reduction	Hemoptysis rates lower	Clear airway visualization	Real-time gating (precise bronchial dose characterization) and daily adaptation
Heart	Lee et al., 2023 [72]	Constraint violations reduced (5% → 0% with adaptation)	No ≥G2 late AE; no G4–5 events	Superior soft-tissue cardiac delineation	Daily adaptive replanning ensuring OAR constraint fulfillment
Great Vessels	Lee et al., 2023 [72]	Constraint violations reduced (21% → 0% with adaptation)	No severe vascular AEs reported	Precise visualization of vascular structures	Daily plan re-optimization to avoid exceeding dose constraints
Lungs	Klaar et al., 2024 [73]	0.10–0.20 Gy MLD reduction (adaptive vs. non-adaptive)	Pneumonitis rates reduced	Functional lung preservation	Ventilation imaging integration
Spinal Cord	Han et al. 2022 [74]	Margin reduction: minimal dose increase despite smaller PTV	Myelopathy remained at 0%	Complete safety maintained	Superior MR visualization
Ribs/Chest Wall	Jasper et al. 2021 [75]	2.5 Gy Dmax reduction with 3 mm margins	Fracture incidence reduced (~20% vs. 40%)	Improved pain outcomes	Real-time localization enables margin reduction

**Table 4:** Conceptual comparison of MR-guided adaptive radiotherapy, CBCT-based adaptive RT, and proton therapy.

	MRgRT	CB-CT Adaptative	Proton therapy
Imaging modality	MRI	Cone-beam CT	CT-based
Beam type	Photon	Photon	Proton
Type of adaptation	Online	Mostly offline	Not widely implemented (complex)
Tumor tracking	Real-time, cine-MRI and gating	Indirect (external surrogates or fiducials)	Limited; motion management is challenging due to interplay effects
Soft-tissue visualization	Good	Limited	Limited
Workflow time	Long (45–90 min)	Short–moderate (15–30 min)	Moderate (20–40 min)
Motion management	Real-time gating and tracking	ITV-based, breath-hold for external gating	Limited (rescanning)
OAR sparing	High	Moderate	Very high
Anatomical changes and range uncertainty	Managed with daily adaptation	Limited adaptation capability	High sensitivity to change (tissue density)
Availability	Limited	Broad	Very limited
Cost	High	Lower	Very high
Evidence level in lung cancer	Emerging	Consolidated	In research

**Table 5:** Key ongoing and planned studies evaluating MR-guided adaptive radiotherapy in lung cancer.

Clinical Trial	Phase	Population	Intervention	Status	Key Relevance
MOMENTUM Registry (NCT04225013)	Prospective registry	All tumor sites including lungs (multi-institutional)	MR-linac RT (all platforms); clinical, dosimetric and imaging data	Ongoing	International evidence base for MRgRT benefits across tumor types
NCT03916419	Phase II	Locally advanced NSCLC (inoperable)	MRgRT + concurrent chemo (curative intent)	Completion, 2025	First MRgRT CCRT curative-intent trial in LA-NSCLC
NCT04925583	Phase I	Ultra-central lung tumors	MR-linac SBRT 10 fx, dose escalation 50–65 Gy + daily ART + gating	Recruiting	Dose-finding for ultra-high-risk anatomical scenarios with daily adaptation
MR-guided SBRT Boost (Burr et al., 2019) [91]	Feasibility	Locally advanced NSCLC (residual PET-avid disease post-CRT)	MRgRT SBRT boost to radioresistant subvolumes after chemoradiotherapy	Published	Improves local control without added AE; targets subvolumes on functional imaging
MR-guided single-fraction SABR [53]	Prospective	Peripheral lung metastases	MR-guided single-fraction SABR	Published 2025	Excellent local control and low AE rates; supports ultra-hypofractionation
STAR-LUNG Study [94]	Phase II	Ultra-central lung tumors	MR-linac SBRT 56 Gy in 8 fx with daily ART + gating	Ongoing	Study to determine feasibility, safety and efficacy of SBRT in ultra-central tumors with MRgRT

ART: adaptive radiotherapy; CRT: chemoradiotherapy; fx: fractions; LA-NSCLC: locally advanced NSCLC; SABR: stereotactic ablative radiotherapy; SBRT: stereotactic body radiotherapy; TITE-CRM: time-to-event continual reassessment method.

## Data Availability

No new data were created or analyzed in this study. Data sharing is not applicable to this article.

## Abbreviations

The following abbreviations are used in this manuscript:

4D-CT	Four-dimensional CT
ADC	Apparent diffusion coefficient
AI	Artificial intelligence
ARS	American Radium Society
ATS	Adapt to shape
ATP	Adapt to position
BEDs	Biologically effective doses
bSSFP	Balanced steady-state free precession
CBCT	Cone-beam CT
CRT	Chemoradiotherapy
DCE	Dynamic contrast-enhanced
DIBH	Deep inspiration breath-hold
DWI	Diffusion-weighted imaging
GTV	Gross tumor volume
IGRT	Image-guided radiotherapy
ICIs	Immune checkpoint inhibitors
ITV	Internal target volume
LC	Local control
MRgRT	Magnetic resonance-guided radiotherapy
MRI	Magnetic resonance image
NSCLC	Non-small-cell lung cancer
NTCP	Normal tissue complication probability
OAR	Organ at risk
oART	Online adaptive radiotherapy
OS	Overall survival
PBT	Proximal bronchial tree
PTV	Planning target volume
RP	Radiation pneumonitis
SCLC	Small-cell lung cancer
SBRT	Stereotactic body radiation therapy
SMART	Stereotactic MR-guided adaptive radiotherapy
